# A Global Perspective on Testing Infants Online: Introducing ManyBabies-AtHome

**DOI:** 10.3389/fpsyg.2021.703234

**Published:** 2021-09-09

**Authors:** Lorijn Zaadnoordijk, Helen Buckler, Rhodri Cusack, Sho Tsuji, Christina Bergmann

**Affiliations:** ^1^Trinity College Institute of Neuroscience, Trinity College Dublin, Dublin, Ireland; ^2^School of English, University of Nottingham, Nottingham, United Kingdom; ^3^International Research Center for Neurointelligence, The University of Tokyo, Tokyo, Japan; ^4^Max Planck Institute for Psycholinguistics, Nijmegen, Netherlands

**Keywords:** global collaboration, replicability, method development, online testing, infancy

## Abstract

Online testing holds great promise for infant scientists. It could increase participant diversity, improve reproducibility and collaborative possibilities, and reduce costs for researchers and participants. However, despite the rise of platforms and participant databases, little work has been done to overcome the challenges of making this approach available to researchers across the world. In this paper, we elaborate on the benefits of online infant testing from a global perspective and identify challenges for the international community that have been outside of the scope of previous literature. Furthermore, we introduce ManyBabies-AtHome, an international, multi-lab collaboration that is actively working to facilitate practical and technical aspects of online testing and address ethical concerns regarding data storage and protection, and cross-cultural variation. The ultimate goal of this collaboration is to improve the method of testing infants online and make it globally available.

## Introduction

Online testing holds vast promise for infant scientists. Conducting developmental research online can foster innovation, impact, and access (e.g., Sheskin et al., 2020) by allowing access to larger, more diverse samples and by creating cost-efficient joint participant databases for easier recruitment. Such possibilities facilitate more reproducible science and ultimately create opportunities to investigate questions that are uniquely accessible by testing diverse populations of infants in their natural home environment and/or in large samples.

The past years have seen a lot of advances on this front: Platforms designed specifically for developmental research (e.g., Scott and Schulz, 2017; Lo et al., 2021a), as well as language-specific participant recruitment initiatives (e.g., ChildrenHelpingScience.com; KinderSchaffenWissen.de) are being developed, and online studies are being conducted (e.g., Scott et al., 2017; Tran et al., 2017; Rhodes et al., 2020).

Although these initiatives provide a useful basis for creating infrastructures for online testing, efforts to overcome language, cultural, and regulatory barriers are still scarce: Practical recommendations and software solutions tend to assume US-based research or, in rare cases, are initiatives within the confines of another country or region. In addition, they do not address all needs of the developmental science community, such as the feasibility of paradigms for online testing (see Scott and Schulz, 2017 for an exception).

In this paper, we aim to provide a global perspective on online infant testing, identifying challenges for the international community that were outside of the scope of previous literature. We then introduce ManyBabies-AtHome, an international, multi-lab effort to improve methods of testing infants online. This collaboration of labs distributed across all populated continents is actively working to facilitate practical and technical aspects of online testing as well as address ethical concerns regarding data storage, data protection, and cross-cultural differences. First, however, we will describe the motivations behind testing infants online.

## The “What” and “Why” of Testing Infants Online

For acquiring data online, several options are available. Apps and games can be used to administer parental questionnaires (e.g., Mayor and Mani, 2019; Chai et al., 2020) or acquire child data (e.g., Frank et al., 2016; Semmelmann et al., 2016; Lo et al., 2021b). Researchers can also conduct experiments while in a video call with the participants (i.e., synchronous testing). This method requires coordination between parent and researcher and imposes the schedule of the researcher as a limiting factor. Therefore, many researchers have turned to asynchronous, browser-based testing, the focal method in this article. In asynchronous testing, parents and their infants participate in experiments at a time that is convenient to them, without an experimenter present. Relevant information (e.g., the infant’s date of birth) is logged and a webcam recording may be made of the infant doing a task on the computer (e.g., looking or touching), with the parent (e.g., reading a book or playing together), or away from the computer (e.g., playing with toys and vocalizing). The data are sent to the experimenter, who can review them at their own time. Asynchronous online testing has several benefits compared to lab-based testing.

The first benefit, participant sample, is 2-fold and pertains both to sample size and sample diversity. Many studies in developmental psychology suffer from low statistical power (Bergmann et al., 2018) due to small sample sizes and limited number of observations per participant (Byers-Heinlein et al., 2021). Online testing has the potential to allow researchers to test larger samples in less time, because (1) participants can participate in parallel; (2) there is no need to schedule the session; and (3) the study is accessible to participants who cannot come to the lab. The latter also means access to more diverse samples (Scott and Schulz, 2017; Rhodes et al., 2020; Cuccolo et al., 2021), such as people who do not live close to research labs or who work full time but who do have access to a computer with an Internet connection. This is important within a country as well as globally. Approximately 12% of the global population is western, educated, industrialized, rich, and democratic (WEIRD), but they make up 80% of participants in psychology experiments (Henrich et al., 2010; see also Nielsen et al., 2017). Conclusions based on these participants may not generalize to the remaining 88% of the population. Online testing, thus, has the potential to improve the robustness of our studies due to well-powered studies, to increase the representativeness of the sample to match the global population, and to increase the ease of testing the generalizability of one’s findings across various demographics.

A second benefit is increased replicability of the experimental protocol. Codified and fully automatized online experiments are easily replicable and extendable: All details related to the design, protocol, instructions, and testing session are specified in sharable and reusable code, materials, and text. This also facilitates collaborations between labs across the world as everyone can use the same protocol and there is no need for specific lab equipment.

A third benefit is reduced cost for the researcher. Running studies online is less labor-intensive and thus cheaper. Especially when testing asynchronously, there is a substantial reduction in the number of hours spent on scheduling and lab visits. Online testing is quick; researchers can in principle recruit and test hundreds of participants in 1day (Berinsky et al., 2012; Casler et al., 2013). Finally, studies can be done at the infant’s convenience, potentially increasing the chance of successful data acquisition, leading to fewer dropouts – whose data acquisition cost time and who may still receive rewards.

The main benefits of online testing thus can be summarized as increased size and diversity of the sample, more replicable and extendable experiments, easier collaboration, and lower cost. The recent pandemic has emphasized an additional benefit: avoiding the risk of infection. This is worth considering more generally, especially when working with physically vulnerable populations, such as infants. The accessibility of the method may also have benefits in clinical settings, for instance for developmental follow-ups. Because of all these benefits, we predict sustained interest in and use of online methods.

## Challenges Associated with Testing Infant Online

Testing infants online also comes with challenges, and many of which are additionally problematic for a global perspective and international collaborations. Software solutions are often inaccessible to large parts of the world due to being optimized for a certain country, law, culture, and/or language. Since current solutions cover North America and some of Europe, the WEIRD bias in participant sampling may be reinforced. It is outside the scope of this paper to discuss all challenges in detail, but in this section, we aim to raise general awareness about the current limits of broadly adopting online testing.

Laws and regulations form the first challenge. Local laws and regulations vastly differ regarding data collection, storage, and sharing. Using US-based platforms might be a problem under the European General Data Protection Regulation (GDPR), for example, because of concerns about who has access to data stored outside of Europe. The vague language and non-static nature of the regulations (see, for instance, the United Kingdom following Brexit) and variability in local interpretations (e.g., Clarke et al., 2019) mean that researchers often are not aware of their options (Greene et al., 2019). This means that Research Ethics Committees (RECs) make decisions based on individual interpretations, causing an additional source of variability. As predicted by Litton (2017), even RECs that are governed under the same law can disagree on consent forms, use of US-based corporate cloud services, reimbursements, and so on. Although this is not specific to online testing, the novelty of the method and technology involved means there is no commonly accepted standard yet, causing a greater degree of unpredictability regarding REC decisions. This makes it difficult to make general recommendations or exchange experiences.

A second challenge pertains to international and cross-cultural data acquisition. Most platforms have been developed in one language (often English). This limits the possibility for global data collection. In addition to the language *per se*, which could be resolved with a translation, there are important cultural and contextual differences that need to be considered, such as a conversion between educational degrees, the formality of language use, and culturally sensitive approach to topics like asking about health and developmental delays. This means that all materials – from landing page to questionnaires – must not only be translated but also be culturally adapted (see Beaton et al., 2002).

A third challenge concerns the accessibility of online testing. Although online testing offers great potential for acquiring larger and more diverse samples, it is important to realize its limitations in terms of accessibility. Online testing relies on access to the Internet, not just for the experiment itself but often also for participant recruitment. Some populations will be easier to recruit *via* Internet advertising and social media presence than others. The best ways of recruiting various subpopulations for online infant testing have yet to be systematically investigated. Moreover, in online testing, the experiment and data quality are determined by participants’ equipment and Internet connection at home. Researchers must consider the study’s equipment and technical requirements, as these may limit data acquisition in certain subpopulations or countries. Online testing has the potential to reach more people but is not yet able to reach everyone. Fortunately, computers, webcams, and Internet connections are becoming increasingly accessible with nearly 50% of the world population using the Internet in 2017, and 16.3% of individuals ranked as having a low income using the Internet in 2017 compared to 2.2% just 10years earlier.^1^

A fourth challenge is obtaining high-quality data. While this challenge is not unique to the issue of globalization, its resolution requires a broad, collaborative perspective. Compared to a lab setting, online testing means less control over factors commonly associated with data quality in infant research. Precise temporal measures and reliance on exact timings can pose challenges (Anwyl-Irvine et al., 2020; Bridges et al., 2020). Furthermore, the parent implicitly takes on the role as co-experimenter regarding, for example, the lighting conditions for any type of video recording, infant positioning, and the presence of distractions. This role of the parent as co-experimenter increases the need for clear and appropriate instructions to ensure good data quality. Furthermore, it may be necessary to expect a higher attrition rate for online studies than lab-based studies due to problems with data quality. Acquiring high-quality data are also a critical prerequisite for automatic coding of participants’ behavior, such as looking behavior from webcam recordings. The latter is still subpar to eye-trackers in the lab; even simply tracking whether an infant is looking to the screen is not accurate enough to be used for infant-controlled procedures (Chouinard et al., 2019). Finally, asynchronous online testing removes certain sources of variability (e.g., differences in protocols between labs), but it likely introduces other sources of noise (e.g., distractors in the environment, increased parental interference, and feasibility to develop a robust online procedure for certain research questions or paradigms). Larger sample sizes and clear parental instructions may counteract some of this noise, but this may not be a solution for all types of research questions. The limitations mentioned in this section should be taken into account when deciding whether to conduct the study in the lab or online.

## INTRODUCING Manybabies-Athome

To bundle the field’s knowledge and advance online testing of infants, a large-scale collaboration, the ManyBabies-AtHome (MBAH) project has been initiated. MBAH is an independent project within the ManyBabies consortium^2^ that aims to contribute to best research practices and universally replicable studies in all sub-fields of developmental science (e.g., language development, learning mechanisms, and social cognition). While previous ManyBabies projects have focused on the validity and replicability of specific findings and theories (see, e.g., Frank et al., 2017; Byers-Heinlein et al., 2020; ManyBabies Consortium, 2020, Visser et al., 2021), the MBAH project focuses on collaborative methods development for global online infant testing.

MBAH advances online testing efforts by (1) assessing the community’s needs and wishes, (2) establishing generally applicable solutions in procedure, documentation, and analysis to make online testing methods accessible and robust across the world and to provide templates and materials for reuse and adaptation, (3) conducting studies to develop and test various paradigms for their suitability and robustness in the context of online infant testing, and (4) collecting and annotating a large dataset of infant gaze data that can be exploited for the development of automatic gaze coding approaches, which are necessary for infant-controlled paradigms.

### Assessing the Needs of the Community

To understand the needs of the community, we conducted two informal surveys of researchers engaged with MBAH in spring 2020.^3^ In the first survey, we asked what methods or paradigms they use in the lab and what methods they would like to use for online testing. The responses indicate that researchers in our consortium are looking for the online version of a variety of paradigms, from preferential looking to parent–child interactions. We further asked the consortium which method or paradigm they would prioritize, which led to a strong support for looking behavior studies and preferential looking in particular. The second survey focused on ethics, data protection, and laws and regulations. At the time, many members of the consortium were subject to GDPR, which had come into effect 2years prior. It is noteworthy that many researchers did not know, at the time of the survey, which options for reimbursements, data storage, and software solutions would be accepted by their RECs or local laws.

### Procedures and Methods

New methods give rise to new questions about procedures. From ethics applications to data analysis, the research community needs to explore the possibilities and will ideally agree on acceptable standards. Direct collaboration within the community *via* MBAH makes this process more efficient and allows researchers to immediately voice their concerns and opinions. This allows us to take cross-cultural considerations, language barriers, and local laws and regulations into account. In collaboration with the consortium, we are making sure that data acquisition and storage meet their local requirements (see also Section “Ethics and Data Protection”). Furthermore, in addition to translating all Web sites visible to the parents and adapting the language use to cultural norms, we work together as a team to make sure the selected stimuli are appropriate and meaningful across cultures and languages (see also Section “Cultural Barriers”). MBAH is thus able to explore the possibilities and evaluate the benefits and downsides of various aspects of online infant testing across the world. Moreover, individual researchers will benefit from the knowledge acquired through MBAH regarding, for instance, the write-up of ethics applications, recruitment of participants, and instructions for parents.

### MBAH Studies

Studies within MBAH are grassroots efforts, where members of the community can propose paradigms to study a research question on any aspect of development and if there is sufficient interest, efforts for joint study design are pooled. The first MBAH studies are efforts designed to suit the unique context of developing online testing methods. MBAH’s initial focus is on studies using looking behavior as the primary measure. We, the MBAH steering committee, opted for asynchronous testing because of its benefits (see Section “The “What” and “Why” of Testing Infants Online”). We further decided, after reviewing several options in summer 2020, to conduct our studies on the LookIt platform (Scott and Schulz, 2017), as this platform is designed specifically for asynchronous testing of infant looking behavior studies and is well tested, supported, and documented. This decision poses certain challenges as it is a US-based platform that uses the commercial cloud for data storage. However, if groups across the world are to be enabled and encouraged to acquire globally representative samples, it will be essential to break down data silos, wherever they may be. We are therefore focusing our efforts on ensuring only de-identified data are shared, while explaining this process to participants, and describing the scientific case for international data sharing to RECs. We also welcome parallel data collection using different platforms, such as Gorilla, but focus our efforts on supporting the use of LookIt. Our hope is that developed materials can be used across platforms.

#### Study 1: Proof-of-Concept Study

This study’s primary goal is to work out general issues of online testing with an international consortium, including practical matters relating to ethics, data protection, and translation/cultural adaptation. As a secondary goal, this first study uses a preferential looking paradigm to assess infants’ preference for static vs. moving images. This preference has been established in the lab (e.g., Shaddy and Colombo, 2004) and therefore makes for a good proof-of-concept study. With this paradigm, we can further assess previous general findings relating to infant looking time, such as whether infants’ looking time decreases with age (Colombo, 2001; Courage et al., 2006). MBAH, like all ManyBabies projects, is committed to transparent and open science and will pre-register the hypotheses and analysis plan for this study.

#### Planned Future Studies

After evaluating whether the procedures developed for the proof-of-concept study are feasible for the developmental science community globally, we will conduct several studies. In part, these will be expanding on Study 1 by addressing other research questions with preferential looking paradigms. Furthermore, MBAH will increase its range of paradigms to include, for instance, a looking-while-listening paradigm that is currently being developed and plans to move toward replications of lab studies. Due to the broad range of expertise within the consortium, the feasibility of these paradigms can be assessed and adjusted at each step in the process. We thus follow the ManyBabies tradition of working toward a consensus-based best test of a phenomenon.

### Automatic Gaze Coding

The data acquired across MBAH studies will be pooled in a rich, annotated dataset. This dataset will serve as the basis for developing and improving automatic gaze coding algorithms. Although webcam-based gaze tracking for adults has made considerable progress (Semmelmann and Weigelt, 2018), tracking infants’ looks are still challenging (Chouinard et al., 2019), causing most labs to resort to manual coding. However, in addition to being labor-intensive, manual coding introduces inter- and intra-rater variability, leading to additional noise, which could be prevented with reliable automated methods. The algorithms will initially be developed for post-hoc offline gaze tracking. However, our goal is to develop online gaze-tracking algorithms, which would allow for infant-controlled paradigms, such as habituation studies.

### Current Challenges for MBAH

#### Ethics and Data Protection

The variability in local laws and the lack of knowledge among researchers regarding the tools and processes that are available to them poses a challenge to composing ethics and data protection protocols that will be acceptable for the RECs of all our consortium members. The main complications relate to data storage and data sharing. We are working on solutions for researchers whose local regulations limit global data storage and sharing (e.g., those based in the EU) to enable them to acquire data too. We aim to obtain umbrella approval for MBAH, which should allow most consortium members to acquire data. Researchers may also apply with their local ethics boards if they need to meet specific criteria that our umbrella approval does not cover. We are committed to finding solutions for all researchers in our consortium.

#### Cultural Barriers

**Figure 1:**
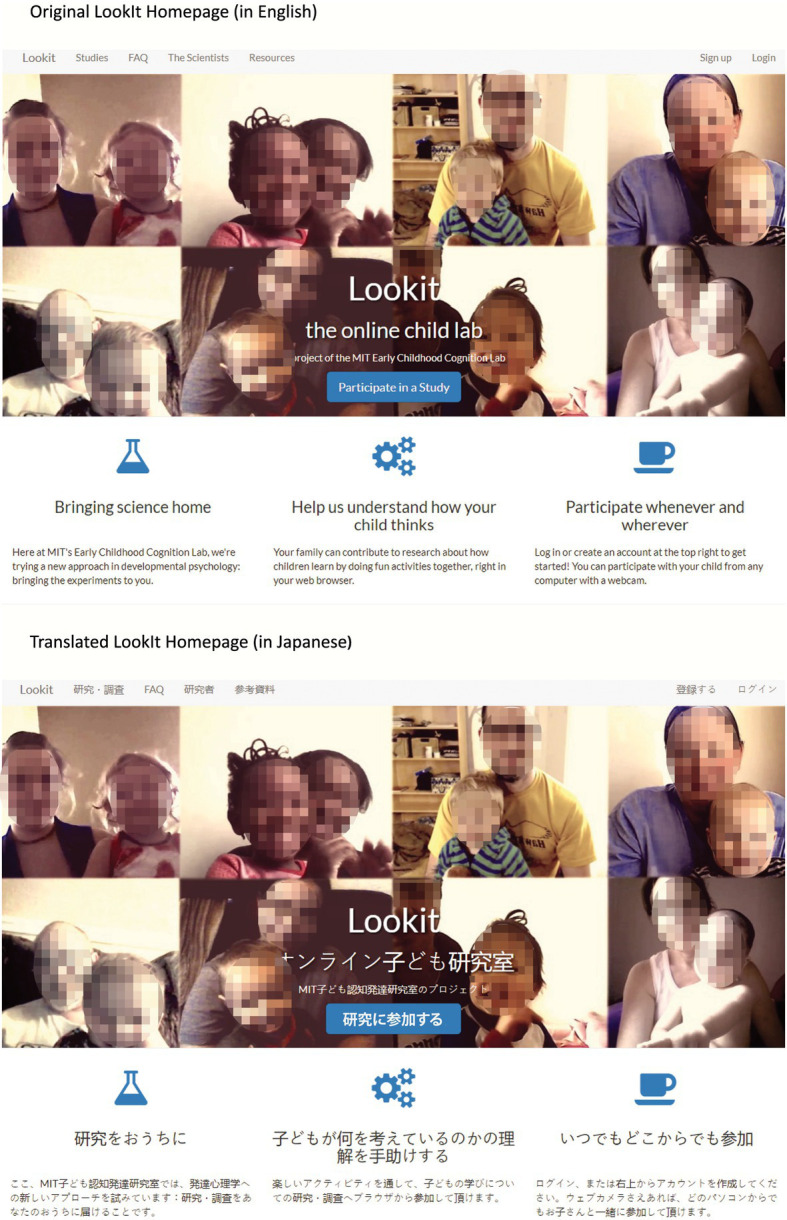
The original LookIt homepage (http://lookit.mit.edu) and an example of one of the translations (Japanese) currently in progress.

Since LookIt, the main platform that will be used for MBAH, is currently targeted at the English-speaking population in the United States, our first objective is to translate this platform into other languages. However, adaptation to other languages and locations goes beyond translation and requires continuous checks for suitability of all questions. In some countries, it is impolite or even illegal to solicit information about infant health (e.g., developmental delays). On the technical side, this requires ensuring that all parts of the platform are contained in files that can be subject to language selection. The translation of the general LookIt pages (such as the homepage, the user’s profile page, and the FAQ) as well as the MBAH study-specific pages of 16 languages is currently in progress (see Figure 1), and MBAH welcomes anyone who speaks English and another language and wants to contribute to our translating effort to join the project. Our translations will hopefully make LookIt a more viable choice for individual researchers outside of the MBAH project as well.

### Contributing to MBAH

MBAH welcomes all researchers and other interested parties to contribute and aims to create an inclusive and diverse environment. Contributors may be at any stage in their career (student to professor), may be from any country, do not have to have participated in earlier ManyBabies projects, do not need to be members of any society, and do not need to know the leadership team to be involved. Interest in potential contribution is free of commitment and can be expressed by email to any of the members of the leadership team (i.e., the authors of this paper), who will send the relevant information. We are keeping track of various types of contributions (according to CRediT principles), which result in authorship on corresponding project papers. For secondary analyses, we aim to openly share anonymized data summaries and where possible (depending on ethical approval and parental consent) share the raw video data.

MBAH plans to also incorporate existing platforms and procedures for data acquisition, processing (e.g., annotation and anonymization), storage, and management. For data acquisition, we have focused on LookIt as our main platform. We recognize, however, that some ethics boards might not approve the use of this US-based platform, in which case data may be acquired elsewhere too. We welcome solutions for any of the above-mentioned data-related processes from research groups, platforms, and companies.

## Conclusion

Online testing offers great potential as a new tool in the developmental scientist’s toolbox as it increases participant diversity, replicability, transparency, and collaborative possibilities and reduces costs for researchers and participants. It has benefits beyond scientific practice too, as it may increase the possibilities for and accessibility of clinical developmental follow-ups. However, despite the rise of platforms and participant databases that make online testing possible, little work has been done to overcome the challenges of making this approach globally available.

Here, we have introduced the international, multi-lab MBAH project. MBAH works to address and resolve the challenges and to create generally applicable solutions in procedure, documentation, and analysis to make online infant testing methods accessible and robust across a range of home environments across the world. Hurdles that are revealed will be resolved in a community-based manner, allowing for rapid and direct input from researchers from different countries and cultures. To accomplish this, we welcome researchers at all levels to join our consortium.

## Data Availability Statement

The original contributions presented in the study are included in the article/Supplementary Material, further inquiries can be directed to the corresponding author.

## Author Contributions

The authors of this article are the full leadership team of MBAH. As such, LZ, HB, RC, ST, and CB have contributed (and are contributing) to the conceptualization, implementation, creating documentation, and funding acquisition related to the MBAH project. LZ wrote the first draft and revision of this article. All authors contributed to the article and approved the submitted version.

## Funding

MBAH is funded by a JST ACT-X grant in the research area “AI powered Research Innovation/Creation” awarded to ST. Moreover, MBAH is facilitated by grants awarded to individual authors: MSCA Individual Fellowship (InterPlay, #891535) awarded to LZ, the ERC Advanced grant (FOUNDCOG, #787981) awarded to RC, and the Jacobs Foundation Fellowship awarded to ST.

## Conflict of Interest

The authors declare that the research was conducted in the absence of any commercial or financial relationships that could be construed as a potential conflict of interest.

## Publisher’s Note

All claims expressed in this article are solely those of the authors and do not necessarily represent those of their affiliated organizations, or those of the publisher, the editors and the reviewers. Any product that may be evaluated in this article, or claim that may be made by its manufacturer, is not guaranteed or endorsed by the publisher.

## Footnotes

^1^ data.worldbank.org

^2^ https://manybabies.github.io/projects/

^3^Since these were informal online questionnaires without informed consent procedures, we cannot publish the actual data and will instead present a qualitative summary of researchers’ responses.
